# *Salvia elegans*, *Salvia greggii* and *Salvia officinalis* Decoctions: Antioxidant Activities and Inhibition of Carbohydrate and Lipid Metabolic Enzymes

**DOI:** 10.3390/molecules23123169

**Published:** 2018-12-01

**Authors:** Olívia R. Pereira, Marcelo D. Catarino, Andrea F. Afonso, Artur M. S. Silva, Susana M. Cardoso

**Affiliations:** 1 Centro de Investigação de Montanha (CIMO), Instituto Politécnico de Bragança, Campus de Santa Apolónia, 5300-253 Bragança, Portugal; oliviapereira@ipb.pt (O.R.P.); andrea@ipb.pt (A.F.A.); 2 QOPNA & LAQV-REQUIMTE, Department of Chemistry, University of Aveiro, 3810-193 Aveiro, Portugal; mcatarino@ua.pt (M.D.C.); artur.silva@ua.pt (A.M.S.S.); 3 Public Health Laboratory of Bragança, Local Health Unit, Rua Eng. Adelino Amaro da Costa, 5300-146 Bragança, Portugal

**Keywords:** sage, phenolic compounds, antioxidant, α-glucosidase, pancreatic lipase, α-amylase, LC-MS analysis

## Abstract

*Salvia elegans* Vahl., *Salvia greggii* A. Gray, and *Salvia officinalis* L. decoctions were investigated for their health-benefit properties, in particular with respect to antioxidant activity and inhibitory ability towards key enzymes with impact in diabetes and obesity (α-glucosidase, α-amylase and pancreatic lipase). Additionally, the phenolic profiles of the three decoctions were determined and correlated with the beneficial properties. The *S. elegans* decoction was the most promising in regard to the antioxidant effects, namely in the scavenging capacity of the free radicals DPPH^•^, NO^•^ and O_2_^•–^, and the ability to reduce Fe^3+^, as well as the most effective inhibitor of α-glucosidase (EC_50_ = 36.0 ± 2.7 μg/mL vs. EC_50_ = 345.3 ± 6.4 μg/mL and 71.2 ± 5.0 μg/mL for *S. greggii* and *S. officinalis,* respectively). This superior activity of the *S. elegans* decoction over those of *S. greggii* and *S. officinalis* was, overall, highly correlated with its richness in caffeic acid and derivatives. In turn, the *S. officinalis* decoction exhibited good inhibitory capacity against xanthine oxidase activity, a fact that could be associated with its high content of flavones, in particular the glycosidic forms of apigenin, scutellarein and luteolin.

## 1. Introduction

*Salvia* genus (*Salvia* spp.), belonging to the Lamiaceae family, comprises more than 900 species that are used for distinct purposes, including the culinary and cosmetic industries or in traditional medicines due to their claimed health benefits [1,2]. Among them, *Salvia officinalis* L., i.e., “common sage” or “Dalmatian sage”, is widely cultivated. These plants usually grow 30–70 cm tall, with a woody stem, whitish beneath and grayish-green above, and with purple-blue flowers up to 3 cm long appearing from early summer to early autumn [1]. Due to its worldwide spread, *S. officinalis* has been the most monitored species in relation to the biological potential of the whole plant as well as of its essential oils and polar extracts. For example, promising results were obtained in clinical studies with aqueous or ethanolic extracts of this *Salvia* species when focused on memory and cognitive functions, pain, and the biochemical profile of glucose and lipids [3]. In addition, in vivo assays in an ear edema induced by croton oil model pointed out the good anti-inflammatory activity of hydroethanolic extracts [4]. Indeed, in vitro experiments demonstrated that its ability to inhibit 5-lipoxygenase activity [5] and to reduce the levels of interleukin 8 (IL-8) [6] might be based on these anti-inflammatory properties. Moreover, several authors also reported the benefits of *S. officinalis* polar extracts towards cell protection in distinct cell-based studies such as in HepG2, HeLa and Caco-2 cell lines, evidencing their possible usage as DNA-protective agents [7,8]. Notably, polar extracts from *S. officinalis* (aqueous, methanolic, ethanolic, and hydroalcoholic) have also been proven to have protective effects against oxidative events [9,10] or oxidative stress-related processes [11,12], as demonstrated in *in chemico*, cell-based or in vivo models [5,10,11,12,13,14,15,16,17].

In addition to *S. officinalis*, other widely distributed sage species such as *Salvia miltiorrhiza* Bge. and *Salvia hispanica* L. were highlighted by their richness in bioactive compounds and their potential health-promoting properties [1,2,18,19,20]. Still, many less-distributed species, including *Salvia greggii* A. Gray and *Salvia elegans* Vahl., remain poorly studied regardless of their broad use for culinary and medicinal purposes. *S. greggii*, also known as “autumn sage”, is originated from Mexico and Texas, although it is currently spread in southwestern United States and Arizona and cultivated in some parts of the world. It grows as a soft, evergreen shrub taller than about 1.2 m, and, similarly to *S. officinalis*, its leaves are green and smooth [21]. Its flowers, which appear between spring to autumn, can be of different colors (red, pink, purple, white or orange) and are characterized by an intense aroma and abundant nectar. In turn, *Salvia elegans* is a species native of Mexico and is currently grown in the United States, Canada, and other regions of the world [22]. It grows as a sub-bush of 1–1.5 m high with a pineapple aroma and flavor, opposite leaves and oval, hairy, elliptic, pale green, and ruby red flowers [23].

*S. greggii* and *S. elegans* are widely used in traditional medicine, particularly in the form of infusions or decoctions, to treat digestive and oral problems (*S. greggii*) [1] or to lower blood pressure and combat central nervous system disorders for anxiety and insomnia (*S. elegans*) [23,24,25]. However, as far as we know, the phytochemical composition and beneficial effects of polar extracts, in particular those related to traditional usage (aqueous), remain unexplored. Regardless of this, *S. greggii* has been screened for terpenic compounds [26,27], and the antigerminative activity of its essential oils [28], while aspects related to polar extracts have not yet been studied. In turn, polar extracts from *S. elegans* have been the focus of some attention, and, in particular, hydroalcoholic extracts have been shown to exhibit antihypertensive, antidepressant, and anxiolytic effects [23,24,25] in in vitro models. Yet, to our knowledge, bioactive constituents of *S. elegans* polar extracts and their ability to counteract oxidative-stress-related events have not been previously elucidated.

Hence, the present study aimed to elucidate the phenolic composition and biological effects of *S. elegans* and *S. greggii* decoctions (mainly focusing on their potential antioxidant activity and inhibitory capacities towards key metabolic enzymes with impact in diabetes and obesity), while comparing the findings to those of the well-known *S. officinalis* species.

## 2. Results and Discussion

### 2.1. Phytochemical Composition

**Figure 1:**
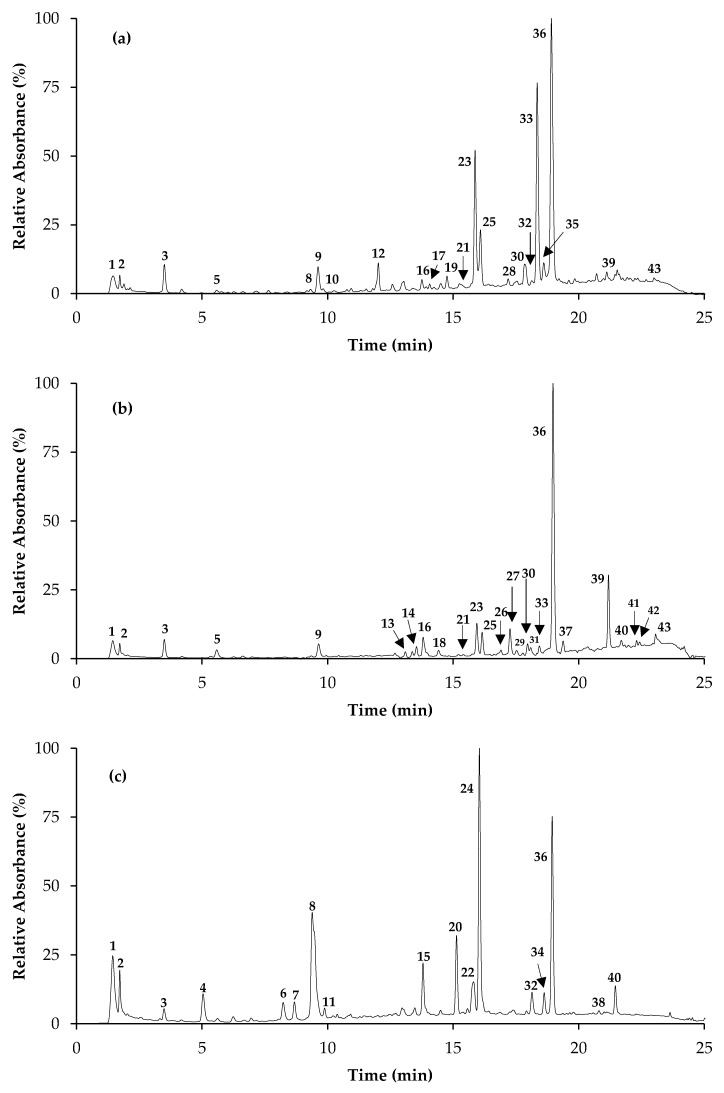
Chromatographic representation of *Salvia officinalis* (**a**), *Salvia elegans* (**b**), and *Salvia greggii* (**c**) decoctions at 280 nm. The numbers in the figure correspond to the UHPLC-DAD-ESI-MS^n^ peaks described in Table 1.

The decoction yields of the three *Salvia* species were approximately 20%, with slightly higher levels observed for *S. elegans* and *S. greggii* in comparison to *S. officinalis* (22.1 ± 2.2% and 22.2 ± 1.5% vs. 19.3 ± 2.3%, respectively). Consistent with their prevalence in *Salvia* plants [1,2,29], caffeic acid derivatives (particularly rosmarinic acid) were dominant compounds in *S. officinalis*, *S. elegans*, and *S. greggii* decoctions, accounting for about one third of the global identified phenolic species (Table 1, Figure 1, Figure S1). Nevertheless, significant differences could be found between extracts. *S. elegans* was distinguished by its richness in caffeic acid derivatives, namely rosmarinic acid (peak 36, [M − H]^−^ at *m*/*z* 359→161, 179), caffeoylrosmarinic acid (peak 39, [M − H]^−^ at *m*/*z* 537→493, 359) and salvianolic acid B (peak 27, [M − H]^−^ at *m/z* 717→519), which overall represented approximately 70% of the total quantified phenolics, while the aqueous extract of *S. greggii* was characterized by high percentages of glycosidic flavones, mostly consisting of luteolin-*O*-hexoside and apigenin-*C*-hexoside (peaks 24 and 20, [M − H]^−^ at *m*/*z* 447 and 431, respectively), representing 33% and 20%, respectively, of total quantified phenolic compounds. Moderate amounts of luteolin-*C*-hexoside (peak 15, [M − H]^−^ at *m*/*z* 447→327, 357), quercetin-*O*-hexoside (peak 22, [M − H]^−^ at *m*/*z* 463→ 301) and two coumaric acid derivatives (peaks 6 and 8, [M − H]^−^ at *m*/*z* 295 and 265, respectively) have also been detected in this extract. Interestingly, none of these compounds were detected in the decoctions of the two other species. Hence, globally, the extracts obtained from *S. elegans* and *S. greggii* species were clearly distinguishable from that of the *S. officinalis,* which was dominated by the *O*-hexuronic form of apigenin ([M − H]^−^ at *m*/*z* 445→269, 48.4 ± 1.3 mg/g extract) and scutellarein ([M − H]^−^ at *m*/*z* 461→285, 13.4 ± 0.6 mg/g extract), in addition to rosmarinic acid (28.3 ± 0.6 mg/g extract). Note that the predominance of apigenin-*O*-glucuronide and rosmarinic acid in this decoction is coherent with the abundance of these two constituents previously reported for polar extracts of this species (e.g., ethanol, methanol, aqueous, hydroalcoholic) [2,30]; however, it is worth noting that this is the first time that scutellarein-*O*-glucuronide was detected in *S. officinalis* extracts, while the previous studies only described its aglycone form.

### 2.2. Biological Activities

#### 2.2.1. Antioxidant Activity

The antioxidant ability of the aqueous extracts obtained from the *Salvia* plants were evaluated in regard to their ability to scavenge free radicals, namely DPPH^•^ (2,2-diphenyl-1-picrylhydrazyl), superoxide (O_2_^•–^), nitric oxide (NO^•^), and peroxyl (RO_2_^•^), and their capacity to reduce Fe^3+^ to Fe^2+^. Furthermore, all the extracts were screened for their potency in inhibiting xanthine oxidase.

Globally, the *S. elegans* decoction was more promising than *S. greggii* in regard to its ability to scavenge free radicals and to reduce Fe^3+^ (Table 2). It presented EC_50_ values about 1.8–2.5 lower than the latter in DPPH^•^, NO^•^, O_2_^•–^, and reducing power tests, and a tendentially higher ability to capture RO_2_^•^. Notably, the *S. elegans* extract also presented tendentially better antioxidant potential than *S. officinalis*, with tendentially reduced EC_50_ values being registered for NO^•^, O_2_^•–^, and reducing power tests, and even three times lower for the DPPH^•^ assay. In addition, one must highlight that the potency of *S. elegans* decoction to counteract DPPH^•^ and NO^•^ was 0.6- and 2.3-fold that of the ascorbic acid, respectively. The only exception was observed for the oxygen radical absorbance capacity (ORAC) assay for which the result observed for *S. officinalis* was better than that for *S. elegans*, although it was not statistically significant.

Table 3 summarizes the correlation coefficients between the amounts of classes of phenolic components found in the *Salvia* decoctions (caffeic acid and derivatives, coumaric acid derivatives, flavones and flavonols) and the data from the distinct biological experiments. According to these results, it is possible to suggest that the superior antioxidant activity of the *S. elegans* decoction is strongly associated with its richness in caffeic acid and derivatives, since correlation factors in DPPH^•^, reducing power, NO^•^, and O_2_^•–^ assays were 0.801, 0.948, 0.986, and 0.844, respectively.

The comparison of the herein gathered data with that previously reported for other solvent-extracts or other *Salvia* species is not an easy task, since methodologic adaptations (e.g., radical precursor concentrations and their producing conditions) cause inevitable changes in EC_50_ values. This difficulty can be partly overcome by the comparison of the extract’s potencies with that of reference compounds. Unfortunately, this approach is often not addressed by the authors. Moreover, there is no universal reference compound for a specific antioxidant assay, and variations in the selected standards are frequent within literature. Regardless of that, one must note that *S. officinalis* polar extracts have been commonly used as a reference for the assessment of antioxidant properties of other less-investigated plants [14,15], showing EC_50_ values in the range of 2.0 to 233.0 μg/mL for the DPPH^•^ assay [5,7,13,14,15,31]. Other ethanolic, methanolic, or aqueous extracts of *Salvia* origin, including those obtained from *Salvia amplexicaulis* [32], *Salvia ringens* [33], *Salvia verbenaca*, *S. sclarea* [34], *Salvia argentea* [15], and *Salvia nemorosa* [35], have been claimed to be good DPPH^•^ scavengers as well, with antioxidant potentials that, in some cases, equal those of the standard compounds (ascorbic acid, butylated hydroxytoluene—BHT, or butylated hydroxyanisole—BHA). Hence, one might conclude that, in agreement with other studies reported for polar extracts of several *Salvia* species, *S. officinalis, S. elegans*, and *S. greggii* decoctions have a high ability to scavenge DPPH^•^, with *S. elegans* showing the most promising activity, followed by *S. greggii* and *S. officinalis.*

Polar extracts obtained from *Salvia* plants have also been previously screened for antioxidant abilities through other assays, although not as frequent as for DPPH^•^. In this context, Hamrouni-Sellami et al. [36] reported that the Fe^3+^ reducing ability of *S. officinalis* methanolic extracts was 6.5-fold less that of ascorbic acid, being in agreement with our results which also pointed to good effectiveness for decoctions of the same species. In general, our results also indicate that the three sage species herein studied possess promising NO^•^ scavenging capacities, as all the extracts had a lower EC_50_ compared to ascorbic acid. Moreover, their activity seems to be superior to that described by Chen and Kang [37] for the methanolic extracts of *Salvia plebeia* (EC_50_ = 216 ± 2.9 μg/mL), albeit that the absence of a reference compound in that study hampered solid conclusions. Furthermore, in our study, *S. elegans* and *S. officinalis* decoctions showed good O_2_^•–^ scavenging capacity, also suggesting that these extracts might be more active than the methanolic extracts of *Salvia splendens* (EC_50_ = 527 μg/mL) [38]. Likewise, decoctions of *S. officinalis*, *S. elegans*, and *S. greggii* showed high capacity to scavenge RO_2_^•^ (336–404 μM TE/mg), which was significantly superior to those previously reported for the aqueous and ethanolic extracts of *S. officinalis* (1143 and 2535 μM TE/g) and that other *Salvia* species (279–4735 μM TE/g). 

Phenolic compounds have been previously reported to counteract the activity of xanthine oxidase (XO) [39,40], i.e., the enzyme that catalyzes the oxidation of hypoxanthine to xanthine, and further catalyzes xanthine to uric acid with a concomitant production of O_2_^•–^, thus contributing to increment of oxidative stress events in cells. As can be observed in Table 2, the decoctions of the three *Salvia* species could effectively inhibit the activity of XO, albeit being less potent than the commercial drug allopurinol (EC_50_ = 55.1–71.8 µg/mL for *Salvia* extracts vs. 0.09 ± 0.01 µg/mL for allopurinol, respectively). Among the extracts, the most powerful was that from *S. officinalis*, a fact that could be related to its richness in apigenin glucuronide or in other flavones (i.e., scutellarein and luteolin glycosides), since these compounds have been described as strong inhibitors of this enzyme [41,42,43,44]. Although the individual effect of the compounds has not been tested by us, the correlation coefficients between the antioxidant assays and the main compounds of each aqueous extract are in good agreement (Table 3). In XO inhibitory assay, the flavones content of the *S. officinalis* decoction was highly correlated (0.901) with its XO inhibition capacity.

#### 2.2.2. Metabolic Enzyme Activity

α-Glucosidase, α-amylase and pancreatic lipase are key digestive enzymes involved in the metabolism of carbohydrates and lipids, which make them important targets for therapeutic control of diabetes and obesity. α-Amylase and α-glucosidase catalyze the hydrolysis of carbohydrates into simple sugars, thus their inhibition retards the digestion of starch and oligosaccharides contributing to the reduction of postprandial increase in plasma glucose levels. In turn, lipase inhibition decreases the digestion of dietary triglycerides, hence reducing the levels of free fatty acids and monoacylglycerols in the intestinal lumen [40,45,46]. In this study, the ability of *S. officinalis, S. elegans*, and *S. greggii* decoctions to inhibit the activity of these three digestive enzymes were assessed through *in chemico* models.

Notably, the inhibitory activities of the three *Salvia* extracts against α-glucosidase were very promising, especially for *S. elegans* and *S. officinalis* (EC_50_ = 36.0 ± 2.7 µg/mL and 71.2 ± 5.0 µg/mL, respectively), demonstrating activities of 9- and 4-times that of the antidiabetic pharmaceutical drug, acarbose, respectively (Table 4). Moreover, despite being less effective than *S. elegans* and *S. officinalis*, *S. greggii* decoction was as effective as acarbose. Hence, our results suggest that the decoctions of *S. elegans*, *S. officinalis*, and *S. greggii* could serve as natural antidiabetic and anti-obesity agents to help in the control of glucose levels through the control of α-glucosidase activity. This hypothesis is also consistent with previous studies that reported identical results for polar extracts of *Salvia* against this enzyme, e.g., hydroethanolic extracts of S. *officinalis* (EC_50_ value of 69.7 µg/mL) [47] and methanolic extracts of *Salvia acetabulosa, S. nemorosa*, and *Salvia chloroleuca* (EC_50_ = 76.9 µg/mL, EC_50_ = 19 µg/mL and EC_50_ = 13.3 µg/mL, respectively) [35,48,49]. In vivo experiments have even demonstrated that the administration of a daily dose of *S. officinalis* methanolic extracts (500 mg/kg body weight) to alloxan-induced diabetic rats caused the inhibition of α-glucosidase activity comparable to that of the administration of acarbose (20 mg/kg bw) [45].

The inhibitory capacity of polar extracts of *Salvia* species towards α-glucosidase have been mostly correlated with their phenolic constituents. In fact, Chen and Kang [37] reported that the inhibition of this enzyme by *S. plebeia* methanolic extracts increased proportionally to their total phenolic content. Moreover, Kocak et al. [50] reported that aqueous and methanolic extracts of *S. cadmica*, both rich in rosmarinic acid, luteolin, and apigenin, had high inhibitory effects towards α-glucosidase and α-amylase. Moreover, several phenolic compounds isolated from *S. miltiorrhiza*, namely, tanshinone IIA, rosmarinic acid, rosmarinic acid methyl ester and salvianolic acid C methyl ester, were reported to be stronger inhibitors of α-glucosidase than acarbose (EC_50_ = 0.042–0.23 μM and EC_50_ = 5.8 μM, respectively) [51]. The flavonoid compounds luteolin-7-*O*-glucoside, luteolin-7-*O*-glucuronide, and diosmetin-7-*O*-glucuronide, isolated from the aerial parts of *S. chloroleuca*, also showed potent α-glucosidase inhibitory effects with EC_50_ values of 18.3, 14.7, and 17.1 μM, respectively, exhibiting an inhibitory effect close to that of acarbose (EC_50_ = 16.1 μM) [49]. Note that rosmarinic acid and caffeoyl rosmarinic acid are two major phenolic components in *S. elegans* decoctions and, based on the mentioned bibliographic data, it is feasible to hypothesize that they might be important contributors for the higher inhibitory ability of this extract compared to the other two. In fact, correlation coefficients determined for the different assays have shown a strong correlation between the results obtained for inhibitory activities on α-glucosidase and the content in caffeic acid and derivatives of the extracts (0.995). Interestingly, high correlation coefficients were also observed between the α-glucosidase and the antioxidant assays (0.976, 0.998, and 0.996 for reducing power, nitric oxide scavenging, and superoxide anion scavenging, respectively; Table 3), suggesting that metabolic and antioxidant effects might possibly be related.

However, regardless the great α-glucosidase inhibitory capacity and the fact that some authors have previously found potential inhibitory capacities in polar extracts of *Salvia*, namely for aqueous and methanolic extracts of *S. cadmica*, as well as for some individual phenolic compounds from *Salvia* origin [49], our results showed no substantial inhibition towards α-amylase up to the concentration of 0.5 mg/mL. Moreover, at 0.2 mg/mL, only *S. greggii* showed an anti-lipase activity higher than 10%. This could possibly be owed to its main phenolic component, i.e., luteolin-7-*O*-glucoside, since its aglycone has been reported to be a good lipase inhibitor [52,53], a hypothesis also supported by the high correlation found between the content of this flavone and the anti-lipase activity (0.930, data not shown). Interestingly, the anti-lipase activity of polar extracts of *Salvia* species has been previously described, namely for the methanolic extract of the leaves of *S. officinalis* (EC_50_ = 94 μg/mL) [54], the methanol extract of *Salvia spinosa* (EC_50_ = 156.2 μg/mL) [55], and methanol extracts of *Salvia triloba* (EC_50_ = 100.8 μg/mL) [56]. Hence, despite data from literature that seems to suggest that at least some polar extracts from *Salvia* species might be promising with respect to their abilities to control the activity of α-amylase and pancreatic lipase, this was not the observed for *S. officinalis, S. elegans* and *S. greggii* decoctions herein studied. 

## 3. Materials and Methods 

### 3.1. Chemicals 

Ethanol, potassium di-hydrogen phosphate, and gallic acid were purchased from Panreac. Dimethylsulfoxide (DMSO), sodium chloride, potato starch, sodium and potassium tartrate, sodium hydroxide, and tris-HCl were purchased from Fisher (Pittsburgh, PA, USA). Fluorescein, 2,2′-azobis(2-amidinopropane)di- hydrochloride (AAPH), sodium nitroprusside, sulfanilamide, and 3,5-dinitrosalicylic acid (DNS) were purchased from Acros Organics (Hampton, NH, USA). Trolox, xanthine oxidase from bovine milk, allopurinol, α-glucosidase from *Saccharomyces cerevisiae*, 4-nitrophenyl α-D-glucopyranoside (pNPG), lipase from porcine pancreas and 4-nitrophenyl butyrate, α-amylase from porcine pancreas, β-nicotinamide adenine dinucleotide (β-NADH), phenazine methosulphate (PMS), nitrotetrazolium blue chloride (NBT), BHA (butylated hydroxyanisole), DPPH radical (2,2-diphenyl-2-picrylhydrazyl), ascorbic acid, and BHT (2,6-di-*tert*-butyl-4-methylphenol) were obtained from Sigma (St. Louis, MO, USA). Calcium chloride and sodium di-hydrogen phosphate were purchased from ChemLab (Eernegem, Belgium). Orlistat was purchased from TCI (Tokyo, Japan), acarbose from Fluka (Bucharest, Romania), xanthine from AlfaAesar (Ward Hill, MA, USA), and *N*-(1-naphthyl)ethylenediamine dihydrochloride from VWR (Radnor, PA, USA). Standard phenolics used for quantitative analysis were obtained from Extrasynthese. Folin-Ciocalteu reagent, Na_2_CO_3_, formic acid, and ethanol were purchased from Panreac (Barcelona, Spain). *n*-Hexane, methanol, and acetonitrile with high performance chromatography (HPLC) purity were purchased from Lab-Scan (Lisbon, Portugal). Water was treated in a Direct-Q^®^ water purification system (Merck Life Science, Darmstadt, Germany). All reagents were of analytical grade or of the highest available purity.

### 3.2. Plant Sampling and Preparation of Extracts

*S. officinallis*, *S. elegans*¸and *S. greggii* were purchased from Ervital (Viseu, Portugal) as a mixture of flowers and leaves, and stems where were cultivated under an organic regime. After collection, the aerial parts were dried in a ventilated incubator at 20 to 35 °C for 3 to 5 days. 

Phenolic compounds were extracted by decoction according the method described by Ferreira et al. [57], with adaptations. A volume of 100 mL of distilled water was added to 5 g of plant material (0.5 mm mesh powder) and the mixture was heated and then boiled for 15 min and filtered under reduced pressure through a G3 sintered plate filters. The resulting filtrated solution was concentrated in a rotary evaporator at 37 °C, followed by defatting with *n*-hexane (1:1 *v/v*). The resulting fraction was frozen, freeze-dried, and kept under vacuum in a desiccator in the dark for subsequent use [58].

### 3.3. Identification and Quantification of Phenolic Compounds

UHPLC-DAD-ESI/MS^n^ analyses of phenolic profiles from *S. officinalis*, *S. elegans*, and *S. greggii* decoctions (5 mg/mL) were carried out on an Ultimate 3000 (Dionex Co., San Jose, CA, USA) apparatus equipped with an ultimate 3000 Diode Array Detector (Dionex Co., San Jose, CA,, USA) and coupled to a Thermo LTQ XL mass spectrometer (Thermo Scientific, San Jose, CA, USA), an ion trap MS equipped with an electrospray ionization (ESI) source, following a method previous described [58]. Control and data acquisition were carried out with the Thermo Xcalibur Qual Browser data system (Thermo Scientific, San Jose, CA, USA). Nitrogen above 99% purity was used, and the gas pressure was 520 kPa (75 psi). The instrument was operated in negative-ion mode with the ESI needle voltage set at 5.00 kV and an ESI capillary temperature of 275 °C. The full scan covered the mass range from *m/z* 100 to 2000. CID–MS/MS and MS^n^ experiments were simultaneously acquired for precursor ions using helium as the collision gas with a collision energy of 25–35 arbitrary units. 

Gradient elution was carried out with a mixture of two solvents. Solvent A consisted of 0.1% (*v/v*) of formic acid in water, and solvent B consisted of acetonitrile, which was degassed and filtrated, using a 0.2 μm Nylon filter (Whatman International, Ltd., Maidstone, England) before use. The solvent gradient used consisted of a series of linear gradients starting from 5% of solvent B and increasing to 23% at 14.8 min, to 35% at 18 min, and to 100% at 21 min over three minutes, followed by a return to the initial conditions.

For quantitative determinations, the parameters of calibration curves, obtained by injection of known concentrations of the exact or structurally-related standard compounds, allowed the calculation of the limits of detection (LOD) and quantification (LOQ) [58].

### 3.4. Antioxidant Activities

#### 3.4.1. DPPH^•^ Scavenging Assay

Extracts capacity for scavenging DPPH^•^ were evaluated following the procedure previously described by Catarino et al. [59]. Ascorbic acid was used as positive control. The concentration of the extract/standard able to scavenge 50% of DPPH^•^ (EC_50_) was determined using linear regression by plotting the percentage of inhibition against the concentration of the extracts.

#### 3.4.2. Ferric Reducing Antioxidant Power (FRAP) Assay

For the reducing power assay, five different concentrations of each extract were prepared (0.05–0.25 mg/mL), and the assay was carried out according to a procedure described previously [59]. BHA was used as the positive control. A linear regression analysis was carried out by plotting the mean absorbance against the concentrations, and the EC_50_ value was determined considering the extract/standard concentration that provided 0.5 of absorbance.

#### 3.4.3. Oxygen Radical Absorbance Capacity (ORAC) Assay

The ORAC assay was performed according to the method previously described by Catarino et al. [60]. In a 96-well plate, 150 μL of fluorescein (10 nM), diluted from the stock solution of 250 μM, with 75 mM phosphate buffer (pH 7.4) were placed together with 25 μL of different trolox concentrations (3.13–25 μM). The same process was repeated for the extracts with final concentrations ranging between 0.4–6.3 μg/mL. For blanks, 25 μL of phosphate buffer was added instead of antioxidant solutions. After 10 min incubation at 37 °C, 25 μL of 2,2′-azobisisobutyramidinium chloride (AAPH) (153 mM) solution was added to each well to reach a final reaction volume of 200 μL. The plate was immediately placed in the plate reader (Biotek, Austria), and fluorescence was monitored every minute over 60 min. The measurement was carried out at 37 °C with automatic agitation for 5 s prior to each reading. Excitation was conducted at 485 nm with a 20 nm bandpass, and emission was measured at 528 nm with a 20 nm bandpass. Six concentration-dependent kinetic curves were obtained for each sample and for trolox as well. The area under the curve (AUC) of the fluorescence decay and Net AUC were calculated according to the following equations (1–3):

(1) $$\mathrm{AUC}=\;1+\sum_{t_{0\;}=\;60\;\min}^{t_{i}\;=\;60\;\min}\frac{R_{i}}{R_{0}},$$

(2) $$\mathrm{Net}\;\mathrm{AUC}\;={\mathrm{AUC}\;}_{\mathrm{sample}}-{\mathrm{AUC}\;}_{\mathrm{blank}},$$

where R_0_ is the fluorescence reading at the initiation of the reaction and R_i_ is the fluorescence reading at the time i. Antioxidant activities (ORAC values) of the extracts were calculated by using the following ratio:

(3) $$\mathrm{ORAC}\;\mathrm{value}=\;\frac{m_{e}}{m_{T}},$$

where *m*_e_ is the slope of the curve of Net AUC vs. extract concentrations, and *m*_T_ is the slope of the curve of Net AUC vs. trolox concentrations. The final results were expressed in μM of trolox equivalents (μM TE) per μg of sample extract.

#### 3.4.4. NO^•^ Scavenging Assay

The NO^•^ scavenging method was adapted from Catarino et al. [60]. Briefly, 100 µL of six different extract concentrations (0–1 mg/mL) were mixed with 100 µL of sodium nitroprusside (3.33 mM in 100 mM sodium phosphate buffer pH 7.4) and incubated for 15 min under a fluorescent lamp (Tryun 26 W). Afterwards, 100 µL of Griess reagent (0.5% sulphanilamide and 0.05% naphthyletylenediamine dihydrochloride in 2.5% H_3_PO_4_) were added to the mixture, which was allowed to react for another 10 min in the dark. The absorbance was then measured at 562 nm, and the percentage of NO^•^ scavenging was calculated using the equation described by Yen and Der Duh [61] as follows:

(4) $$\%\;{\mathrm{NO}}^{•}\;\mathrm{scavenging}=\;\frac{A_{c}-{\;A}_{e}}{A_{c}}\times 100,$$

where A_c_ is the absorbance of the control (without extract addition) and A_e_ is the absorbance of the extract. Ascorbic acid was used as the reference compound. The concentration of the extract/standard able to scavenge 50% of NO^•^ (EC_50_) was then calculated by plotting the percentage of inhibition against the extract concentrations.

#### 3.4.5. Superoxide Anion (O_2_^•–^) Scavenging Assay

The O_2_^•–^ scavenging method was carried out according to the method described by Catarino et al. [60]. Briefly, in a 96-well plate, 75 µL of six different sample concentrations (0.0–250 µg/mL) were mixed with 100 μL of β-NADH (300 μM), 75 μL of NBT (200 μM), and 50 μL of PMS (15 μM). After 5 min, the absorbances at 560 nm were recorded and the scavenging activity of superoxide radicals was calculated according to Equation (4). Gallic acid was used as the reference compound. The concentration of the extract/standard able to scavenge 50% of O_2_^•–^ (EC_50_) was determined using linear regression by plotting the percentage of inhibition against the concentration of the extracts.

#### 3.4.6. Inhibition of Xanthine Oxidase Activity

Inhibition of xanthine oxidase activity was carried out following the method described by Filha et al. [62], with slight modifications. Briefly, in a 96-well plate, 40 µL of extract concentrations (0–2 mg/mL) were mixed with 45 µL of sodium dihydrogen phosphate buffer (100 mM, pH 7.5) and 40 µL of enzyme (5 mU/mL). After 5 min incubation at 25 °C, the reaction was started with the addition of 125 µL of xanthine (0.1 mM dissolved in buffer) and the absorbance at 295 nm was measured every 45 s over 10 min at 25 °C. The inhibitory effects towards xanthine oxidase activity was calculated as follows:

(5) $$\%\;\mathrm{inhibiton}=\;\frac{m_{c}-\;m_{e}}{m_{c}}\times 100,$$

where *m*_c_ is the slope of the straight-line portion of the curve generated by the control (no inhibitor) and *m*_e_ is the slope of the straight-line portion of the curve generated by each extract. Allopurinol was used as a positive control of inhibition. The concentration of the extract/standard able to inhibit 50% (EC_50_) of the activity of the enzyme was determined using linear regression by plotting the percentage of inhibition against the concentration of the extracts.

### 3.5. Inhibition of Enzymatic Activities

#### 3.5.1. Inhibition of α-Glucosidase Activity

Inhibition of α-glucosidase activity was measured following the method described by Neto et al. [63], with slight modifications. In short, 50 µL of different extract concentrations (0–2 mg/mL, in 50 mM phosphate buffer pH 6.8) were mixed with 50 µL of 6 mM 4-nitrophenyl α-d-glucopyranoside (pNPG), dissolved in deionized water. The reaction was started with the addition of 100 µL of α-glucosidase solution, and the absorbance was monitored at 405 nm every 60 s for 20 min at 37 °C. The inhibitory effects towards α-glucosidase activity was calculated as in Equation (5). Acarbose was used as a positive control of inhibition. The concentration of the extract/standard able to inhibit 50% (EC_50_) of the activity of the enzyme was determined using linear regression by plotting the percentage of inhibition against the concentration of the extracts.

#### 3.5.2. Inhibition of α-Amylase Activity

Inhibition of α-amylase activity was measured according to Wickramaratne et al. [64], with slight modifications. Briefly, 200 µL of extract six different extract concentrations (0–2 mg/mL) dissolved in 20 mM phosphate buffer (pH 6.9, containing 6 mM of NaCl) were added to 400 µL of a 0.8% (w/v) starch solution in the same phosphate buffer, and the mixture was incubated for 5 min at 37 °C. The reaction was then started with the addition of 200 µL of α-amylase solution, and after 5 min of incubation, 200 µL of the reaction mixture was collected and immediately mixed with 600 µL of DNS reagent (10 g/L of 3,5-dinitrosalicylic acid, 300 g/L of potassium and sodium tartrate tetrahydrate, and 0.4 M NaOH) to stop the reaction. A second aliquot of 200 µL was further collected 15 min later and mixed with DNS reagent as well. Samples were then boiled for 10 min, and, once they had cooled, 250 µL were transferred to each well in a 96-well microplate for absorbance reading at 450 nm. Blank readings (no enzyme) were then subtracted from each well and the inhibitory effects towards α-amylase activity was calculated as follows:

(6) $$\%\;inhibiton=\;\frac{\Delta Abs_{c}-\;\Delta Abs_{e}}{\Delta Abs_{c}}\times 100,$$

where ΔAbs_c_ is the variation in the absorbance of the negative control and ΔAbs_e_ is the variation in the absorbance of the extract. Acarbose was used as a positive control of inhibition. 

#### 3.5.3. Inhibition of Pancreatic Lipase Activity

The lipase activity was measured according to the procedure described by Neto et al. [63], with slight modifications. The reaction mixture was prepared in a microtube by mixing 55 µL of five different concentrations of extract (0–2 mg/mL) dissolved in tris buffer 100 mM (pH 7.0) with 467.5 µL of tris-HCl (100 mM, pH 7.0, containing 5 mM of CaCl_2_) and 16.5 µL of enzyme. The reaction was started by adding 11 µL of 20 mM 4-nitrophenyl butyrate diluted in DMSO. Final DMSO concentration in the reaction mixture did not exceed 2%. The reaction mixture was then quickly transferred to a 96-well plate and incubated for 35 min at 37 °C while the absorbance was being measured every 60 s at 410 nm. The inhibitory effects towards pancreatic lipase activity was calculated as in Equation (5). Orlistat was used as a positive control of inhibition.

### 3.6. Statistical Analysis

All data are presented as mean ± standard deviations from three independent assays performed at least in duplicate. One-way analysis of variance (ANOVA) followed by Tukey´s test was used to detect any significant differences among different means. Correlation analyses were performed using a two-tailed Pearson’s correlation test. A *p*-value less than 0.05 was assumed as significant. The results were analyzed using GraphPad Prism 6 (GraphPad Software, La Jolla, CA, USA) and SPSS v 23.0 (Statistical Package for the Social Sciences).

## 4. Conclusions

This work clarifies the antioxidant properties of *S. elegans, S. greggii*, and *S. officinalis* decoctions as well as their inhibition towards the activity of carbohydrate and lipid metabolic enzymes, highlighting possible correlations with their phenolic components. It was shown that among the three plants, *S. elegans* decoctions were the most promising regarding antioxidant activity and inhibitory potential against α-glucosidase, a fact that might be related to its richness in caffeic acid and its derivatives. In turn, despite all the three decoctions of *Salvia* species could effectively inhibit the activity of xanthine oxidase, one should highlight the superior inhibitory capacity of *S. officinalis*, which is possibly associated with the presence of flavones. In conclusion, similarly to the well-known *S. officinalis* species, *S. elegans* and *S. greggii* are a valuable source of natural metabolites and could be used for commercial applications in novel functional foods or pharmaceutical ingredients targeting diabetes and obesity prevention.

## Figures and Tables

**Table 1:** Phytochemical composition of *S. officinalis*, *S. elegans*, and *S. greggii* decoctions determined by UHPLC-DAD-ESI-MS^n^.

NP	RT (min)	λmax	[M − H]^−^	ESI-MS^2^ Main Fragments	Compound	S. off *	S. ele *	S. gre *
1	1.5	275	149	103, 87, 131, 59	2,4-DimethylBA	4.1 ± 0.2	5.1 ± 0.1	7.3 ± 0.2
2	1.7	205	191	111, 173	Quinic acid	0.6 ± 0.1	0.6 ± 0.1	0.5 ± 0.01
3	3.6	280	197	179, 73, 153	Danshensu	D	D	D
4	5.0	290, 324	353	191, 179, 135, 173	*cis* 3-*O*-CQA	-	-	3.1 ± 0.03
5	5.7	220, 278	137	109, 93, 119	HydroxyBA	D	2.0 ± 0.1	-
6	8.3	313	295	163	*p*-Coum Ac Pent	-	-	0.3 ± 0.02
7	8.8	290, 325	353	191, 179	*trans*-5-*O*-CQA	-	-	1.9 ± 0.1
8	9.4	313	265	177, 149, 119	Coumaric Ac Der	-	-	2.9 ± 0.05
		ND	325	163, 119	Caff Hex	D	-	-
9	9.7	290, 323	179	135	CaffAc	1.8 ± 0.04	1.5 ± 0.02	-
10	9.8	314	325	265, 235, 163	Coum Hex	D	-	-
11	9.9	255, 265, 350	625	463, 301	Querc diHex	-	-	D
12	12.1	271, 336	593	473, 503, 353	Api-6-*C*-Glc-7-*O*-Glc	4.3 ± 0.1	-	-
13	13.1	291, 311	637	351, 285, 193	Ferulic Ac Der	-	D	-
14	13.5	274	571	527, 483, 439, 373	YA E (isom1)	-	1.9 ± 0.1	-
15	13.9	256, 267, 345	447	327, 357	Lut-*C*-Hex	-	-	4.6 ± 0.09
16	13.9	281, 345	477	301, 373, 343, 397	Hydroxy-Lut-GlcA	D	1.9 ± 0.2	-
17	14.1	276	571	527, 439, 553, 483	YA E (isom2)	D	-	-
18	14.4	269, 304	473	311, 293, 179, 135	Cichoric acid	-	1.6 ± 0.07	-
19	14.8	267, 345	621	351, 269	Api-diGlcA	4.6 ± 0.3	-	-
20	15.2	268, 336	431	311, 341, 269	Api-*C*-Hex	-	-	15.7 ± 0.3
21	15.4	274	555	313, 357	SA K	1.6 ± 0.2	-	-
			571	527, 553, 509, 329	YA E (isom3)	D	-	-
22	15.8	255, 350	463	301	Querc-*O*-Hex	-	-	2.7 ± 0.2
23	15.9	280, 333	461	285	Scut-*O*-GlcA	13.4 ± 0.6	3.9 ± 0.1	-
24	16.0	255, 265, 348	447	285	Lut-7-*O*-Glc	-	-	26.1 ± 0.9
25	16.1	255, 266, 345	461	285	Lut-7-*O*-GlcA (isom1)	8.4 ± 0.3	5.1 ± 0.3	-
26	16.9	271, 306	521	359, 197, 179, 135	Salviaflaside	-	D	-
27	17.2	278	717	519, 475, 537, 339	SA B (isom1)	-	7.8 ± 0.4	-
28	17.3	279	571	527, 553, 329	YA E (isom4)	0.9 ± 0.1	-	-
29	17.7	279	717	537, 519, 339, 295	SA B (isom2)	-	1.7 ± 0.6	-
30	17.9	268, 334	577	269	Api-rut	4.5 ± 0.1	D	-
		283	719	359, 539, 521, 341	Sagerinic acid	6.0 ± 0.3	D	-
31	18.1	271, 304	717	519, 607, 339, 537	SA B (isom3)	-	1.7 ± 0.1	-
32	18.1	269, 329	431	269	Api-Hex	D	-	3.4 ± 0.2
33	18.4	267, 337	445	269, 175	Api-GlcA	48.4 ± 1.3	3.2 ± 0.5	-
34	18.6	254, 266, 345	533	489, 447, 433	Lut malonyl Hex	-	-	D
35	18.6	270, 291, 326	717	555, 519, 475, 357	SA B (isom4)	D	-	-
36	19.0	218, 290, 328	359	161, 179, 197, 223	RA	28.3 ± 0.6	35.5 ± 0.8	10.9 ± 0.2
37	19.2	269, 307, 343	461	285	Lut-*O*-GlcA (isom2)	-	1.8 ± 0.1	
38	20.8	293, 328	373	343, 329, 311, 179	Methyl rosmarinate	-	-	D
39	21.2	290, 333	537	493, 359, 375	CaffRA/SA I (isom1)	1.2 ± 0.1	17.9 ± 0.1	-
40	21.4	293, 328	329	285, 314, 311, 161	CaffAc derivative	-	-	5.0 ± 0.03
		239, 285, 330	537	456, 493, 375, 359	CaffRA (isom2)	-	1.3 ± 0.04	-
41	22.1	295, 325	713	493, 359, 375	CaffAc der	-	D	-
42	22.3	280	537	456, 493, 359, 161	CaffRA (isom3)	-	0.7 ± 0.04	-
43	23.0	289, 327	717	519, 357, 555	SA B isomer	D	2.4 ± 0.04	-
				*Caffeic acid and derivatives*		39.8 ± 0.9	74.1 ± 0.5	20.8 ± 0.3
				*Coumaric acid derivatives*		-	-	3.2 ± 0.06
					*Flavones*	83.5 ± 2.3	15.9 ± 0.9	49.7 ± 1.3
					*Flavonols*	-	-	2.7 ± 0.2

NP—Number of peak represented in Figure 1; D—Detected; Ac—acid; Api- Apigenin; BA—Benzoic acid; CaffAc—Caffeic acid; Caff—Caffeoyl; CQA—Caffeoylquinic acid; Coum—Coumaroyl; Der—Derivative; Glc—Glucoside; GlcA—Glucuronide; Hex—Hexoside; Lut—Luteolin; Pent—Pentoside; Querc—Quercetin; Rut—Rutinoside; RA—Rosmarinic acid; SA—Salvianolic acid; *S. off*—*S. officinalis*; *S. ele*—*S. elegans*; *S. gre*—*S. greggii*; Scut—Scutellarein; YA—Yunnaneic acid; * values expressed as mg/g of extract.

**Table 2:** Antioxidant properties of *S. officinalis, S. elegans*, and *S. greggii* decoctions.

	*S. officinalis*	*S. elegans*	*S. greggii*	Standard
DPPH^•^ (EC_50_ μg/mL) ^(1)^	34.8 ± 3.3^a^	10.7 ± 2.1^b^	21.1 ± 2.5^c^	6.69 ± 0.7^b^
Reducing Power (EC_50_ μg/mL) ^(2)^	40.0 ± 11.2^a^	31.3 ± 5.0^a,c^	77.9 ± 5.6^b^	16.30 ± 1.5^c^
NO^•^ (EC_50_ μg/mL) ^(1)^	118.2 ± 16.4^a^	91.5 ± 14.5^a^	167.8 ± 23.9^b^	212.1 ± 9.7^c^
O_2_^•–^ (EC_50_ μg/mL) ^(3)^	32.8 ± 0.6^a^	30.6 ± 1.3^a^	61.7 ± 3.4^b^	7.8 ± 0.5^c^
ORAC (μM TE/mg ext) ^(4)^	404.4 ± 1.80^a^	373.1 ± 28.1^a^	335.6 ± 69.6^a^	-
Xanthine oxidase (EC_50_ μg/mL) ^(5)^	55.1 ± 10.6^a^	71.8 ± 3.8^b^	70.1 ± 4.0^a,b^	0.09 ± 0.01^c^

^(1)^ Ascorbic acid was used as the reference compound. ^(2)^ Amount of extract able to provide 0.5 of absorbance by reducing 3.5 μM Fe^3+^ to Fe^2+^. Butylated hydroxyanisole (BHA) was used as a reference compound. ^(3)^ Gallic acid was used as the reference compound. ^(4)^ TE—Trolox Equivalent. ^(5)^ Allopurinol was used as the reference compound. Mean values ± SD; statistical analysis was performed by one-way ANOVA followed by Tukey’s test. In each line, different letters mean significant differences (*p* < 0.05).

**Table 3:** Correlation coefficients between the amounts of phenolic components found in the *Salvia* decoctions (caffeic acid and derivatives, coumaric acid derivatives, flavones and flavonols) and the data from the distinct biological experiments.

	DPPH	RP	ORAC	NO	O2	XO	AG	L
Flavones	− 0.971	− 0.357	0.454	− 0.498	− 0.123	0.901	− 0.551	− 0.367
Flavonols	− 0.239	− 0.934	− 0.891	− 0.868	− 0.992	− 0.434	− 0.835	0.930
CafAcD	0.801	0.948	0.400	0.986	0.844	− 0.237	0.995	− 0.485
CouAcD	− 0.239	− 0.934	− 0.891	− 0.868	− 0.992	− 0.434	− 0.835	0.930
DPPH		0.570	− 0.228	0.690	0.356	− 0.771	0.734	0.134
RP			0.670	0.988	0.971	0.084	0.976	− 0.738
ORAC				0.547	0.829	0.796	0.493	− 0.995
NO					0.922	− 0.071	0.998 *	− 0.624
O2						0.321	0.996	− 0.878
XO							− 0.134	− 0.735
AG								− 0.574

Values expressed as Pearson correlation coefficient *R*; AG—α-glycosidase inhibitory activity; CafAcD—caffeic acid and derivatives; CouAcD—coumaric acid derivatives; DPPH—DPPH radical scavenging activity; L—lipase inhibitory activity; NO—nitric oxide radical scavenging capacity; ORAC—oxygen radical absorbance capacity; O2—superoxide anion scavenging activity; RP—reducing power potential; XO—xanthine oxidase inhibitory activity; * *p* < 0.05.

**Table 4:** Enzyme inhibitory properties of *S. officinalis, S. elegans*, and *S. greggii* decoctions.

	*S. officinalis*	*S. elegans*	*S. greggii*	Standard
α-Glucosidase (EC_50_ μg/mL) ^(1)^	71.2 ± 5.0^a^	36.0 ± 2.7^b^	345.3 ± 6.4^c^	357.8 ± 12.3^c^
α-Amylase ^(2)^	-	-	6.5 ± 3.0	0.7 ± 0.2
Pancreatic lipase ^(3)^	4.6 ± 3.6^a^	8.2 ± 0.3^a^	14.4 ± 7.4^a^	1.8 ± 0.4

^(1)^ Acarbose was used as standard. ^(2)^ Results are expressed as percentage (%) inhibition at the concentration of 0.5 mg/mL (*Salvia* decoctions) or as EC_50_ (μg/mL), for the reference compound acarbose. ^(3)^ Results are expressed as percentage (%) inhibition at the concentration of 0.2 mg/mL (*Salvia* decoctions) or as EC_50_ (ng/mL), for the reference compound orlistat. In each line, different letters mean significant differences (*p* < 0.05).

## Supplementary Materials

Supplementary Materials can be found in a separate file: Figure S1: UV spectra of the main peaks identified in *S. elegans, S. greggii*, and *S. officinalis* decoctions.
