# Supplementary material for: Salvia elegans, Salvia greggii and Salvia officinalis Decoctions: Antioxidant Activities and Inhibition of Carbohydrate and Lipid Metabolic Enzymes
Source: Molecules. 2018 Dec 1;23(12):3169. doi: 10.3390/molecules23123169 (PMC6321363; doi:10.3390/molecules23123169)
Supplement: Supplementary file 1 [file molecules-23-03169-s001.pdf]

Salvia elegans, *Salvia greggii* and *Salvia officinalis* Decoctions: Antioxidant Activities and Inhibition of Carbohydrate and Lipid Metabolic Enzymes

Olívia R. Pereira [1], Marcelo D. Catarino [2], Andrea F. Afonso [1,2,3], Artur M. S. Silva [2] and Susana M. Cardoso [2,*]

[1] Centro de Investigação de Montanha (CIMO), Instituto Politécnico de Bragança, Campus de Santa Apolónia, 5300-253 Bragança, Portugal; oliviapereira@ipb.pt (O.R.P.); andrea@ipb.pt (A.F.A.)
[2] QOPNA & LAQV-REQUIMTE, Department of Chemistry, University of Aveiro, 3810-193 Aveiro, Portugal; mcatarino@ua.pt (M.D.C.); artur.silva@ua.pt (A.M.S.S.)
[3] Public Health Laboratory of Bragança, Local Health Unit, Rua Eng. Adelino Amaro da Costa, 5300-146 Bragança, Portugal
* Correspondence: susanacardoso@ua.pt; Tel.: +351-234-370-360; Fax: +351-234-370-084

Supplementary Materials:

Peak 20

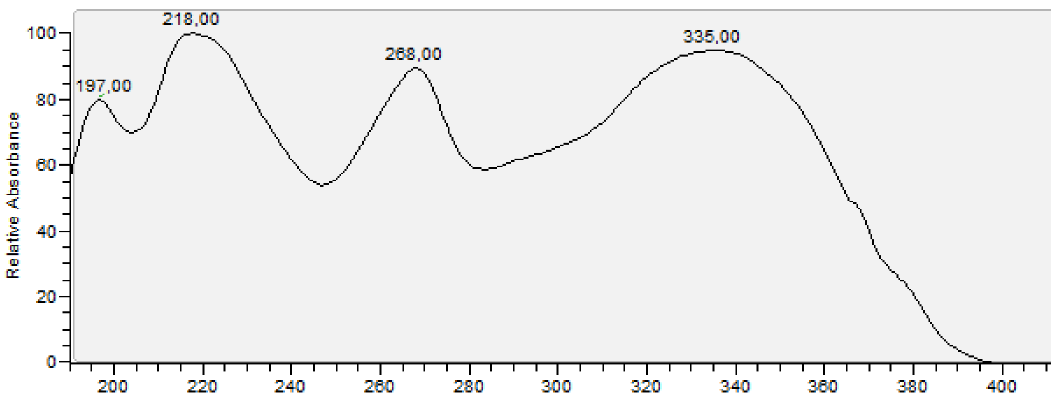

Peak 23

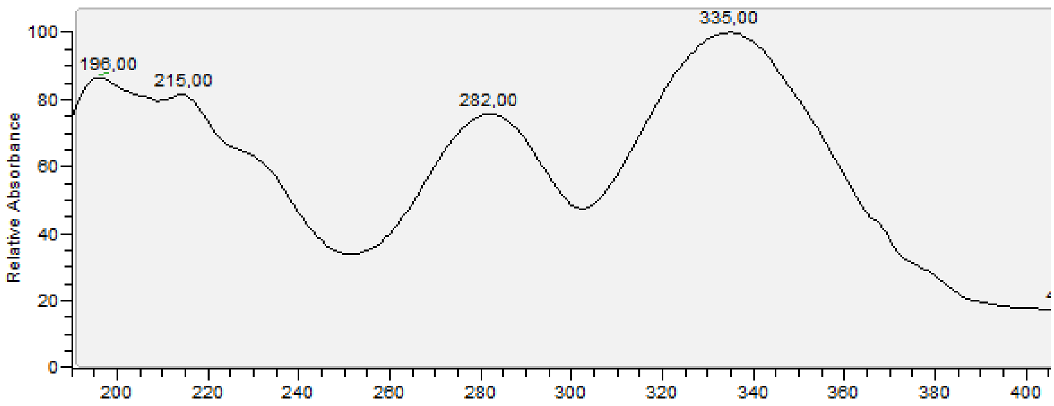

Peak 24

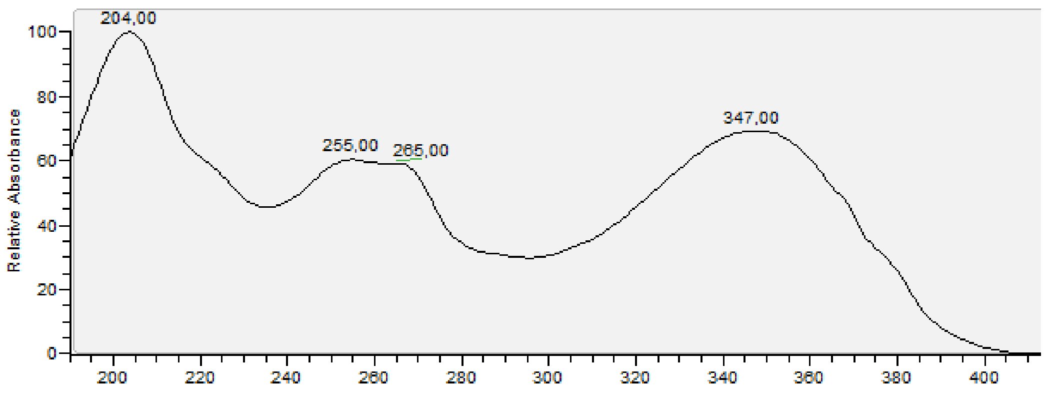

Peak 25

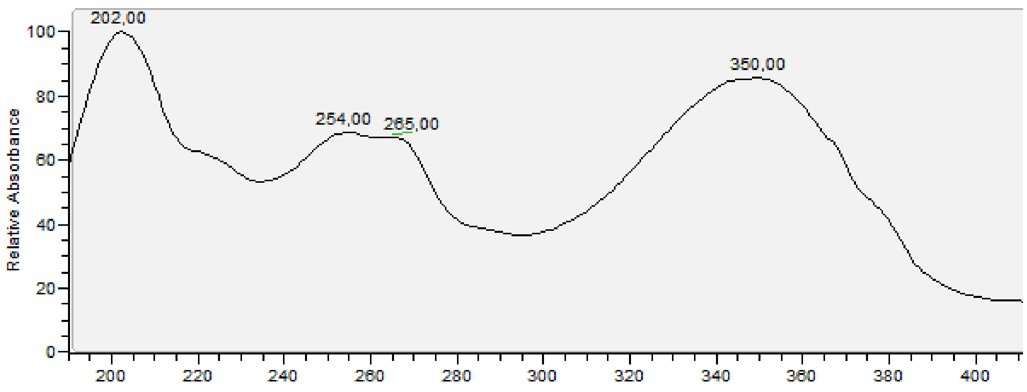

Peak 33

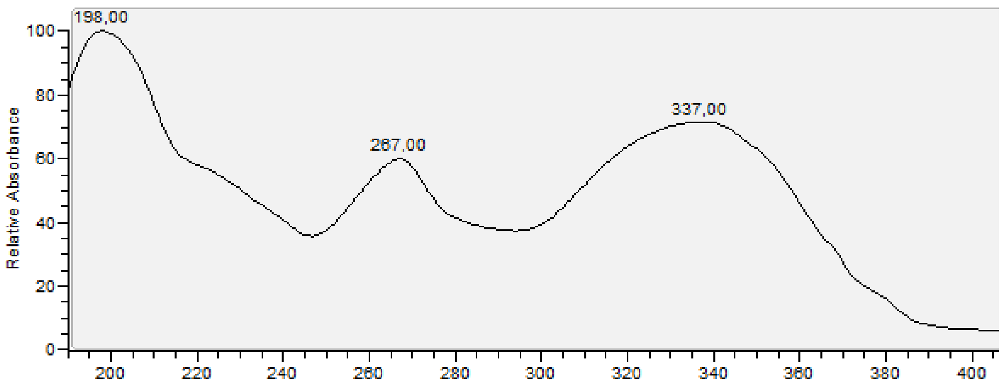

Peak 36

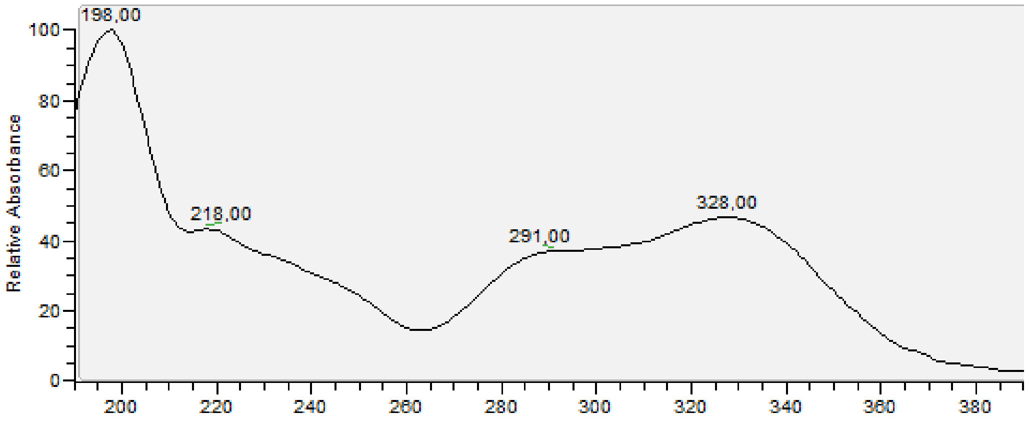

Peak 39

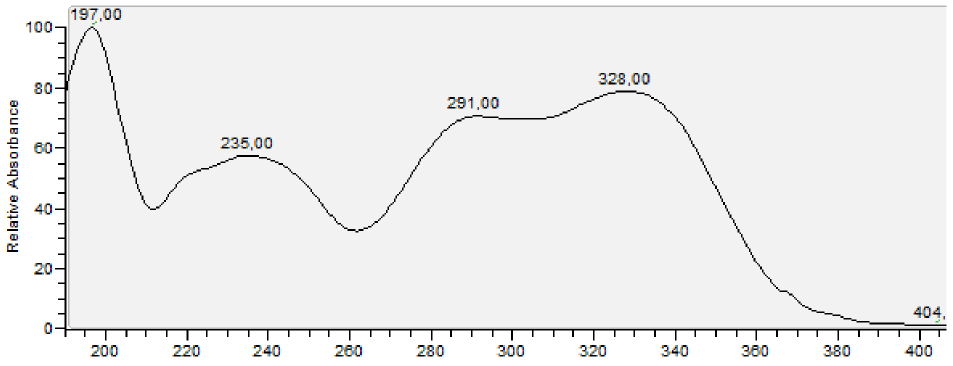

Figure S1. UV spectra of the main peaks identified in *S. elegans, S. greggii*, and *S. officinalis* decoctions. Numbers correspond to the UHPLC-DAD-ESI-MS^n peaks described in Table 1.
